# Circulating tumor cells in the differential diagnosis of adnexal masses

**DOI:** 10.18632/oncotarget.20428

**Published:** 2017-08-24

**Authors:** Dong Hoon Suh, Miseon Kim, Jin Young Choi, Jiyoon Bu, Yoon-Tae Kang, Byung Su Kwon, Banghyun Lee, Kidong Kim, Jae Hong No, Yong-Beom Kim, Young-Ho Cho

**Affiliations:** ^1^ Department of Obstetrics and Gynecology, Seoul National University Bundang Hospital, Seongnam, Korea; ^2^ Department of Bio and Brain Engineering, Korea Advanced Institute of Science and Technology (KAIST), Daejeon, Korea; ^3^ Department of Obstetrics and Gynecology, Pusan National University Hospital, Busan, Korea; ^4^ Department of Obstetrics and Gynecology, Hallym University Kangdong Sacred Heart Hospital, Seoul, Korea

**Keywords:** ovarian neoplasm, circulating tumor cell, preoperative period, differential diagnosis, early stage cancer

## Abstract

The aim of this study was to evaluate circulating tumor cell (CTC) detection in the differential diagnosis of adnexal masses. A total of 87 preoperative women with an indeterminate adnexal mass were prospectively enrolled. Preoperative diagnostic modalities including CTC detection, risk of ovarian malignancy algorithm, risk of malignancy index, and computed tomography or magnetic resonance imaging were compared. Forty-three (49.4%) benign tumors, 13 (14.9%) borderline malignant masses, and 31 (35.7%) cancers were pathologically confirmed. Forty-nine (56.3%) cases were positive for CTCs: 19/43 (44.2%) benign, 10/10 (100%) early-stage, and 14/21 (66.7%) advanced-stage cancer. CTC detection had sensitivities of 77.4%, 100%, and 100% for benign vs. all stage cancer (n = 74), benign vs. stage I–II cancer (n = 53), and benign vs. stage I cancer (n = 49), respectively. CTC detection had a specificity of 55.8% across all comparisons. The sensitivities of the other modalities assayed were decreased in stage I–II cancer and stage I cancer vs. benign masses. Receiver operating characteristic curves showed that CTCs, of which the area under the curve was modest in all stage cancer (0.655), had the widest area under the curve among the evaluated modalities in stage I–II cancer and stage I cancer (0.768 for both). In conclusion, our study findings suggest that preoperative CTCs could have a substantial role in differentiating early stage cancer from benign tumors for adnexal masses.

## INTRODUCTION

About 22,440 cases of ovarian cancer will be newly diagnosed in the United States in 2017 [1]. The incidence of ovarian cancer in Korea has increased gradually from 3.2 to 4.8 per 100,000 females between 2004 and 2014 [2]. Intraoperative rupture of the ovary-confined stage IA cancer results in an upstaging to IC1. This iatrogenic upstaging makes the patient receive otherwise unnecessary chemotherapy in order to minimize the risk of tumor recurrence [3]. Upstaging often happens when ovarian cancer is preoperatively misdiagnosed as a benign tumor and tumor cells migrate during surgery.

Many studies have examined the modalities currently used for preoperatively discriminating adnexal tumors, such as serum biomarkers (cancer antigen-125 [CA-125] and the risk of ovarian malignancy algorithm [ROMA]), imaging studies (computed tomography [CT], magnetic resonance imaging [MRI], and positron emission tomography [PET]), or modalities combining the two (risk of malignancy index [RMI]). However, as these methods provide an inadequate level of sensitivity and specificity, there remains an unmet medical need for a more convenient and non-invasive method with better diagnostic performance.

Circulating tumor cells (CTCs) are viable tumor cells disseminated from the site of disease in metastatic and/or primary neoplasms that can be isolated from the peripheral blood [4]. CTC detection is convenient and minimally invasive. There are multiple lines of literature reporting prognostic significance of CTC as well as the clinical usefulness of CTC as a therapy monitoring tool in various kinds of cancer including breast, colorectal, lung, and prostate cancers [5–9]. Although there has been a growing interest in evaluating the clinical value of CTCs in ovarian cancer, almost all of the relevant studies have explored CTC detection as a prognostic biomarker for tumor burden, risk of residual disease after debulking surgery, and treatment response [4]. To the best of our knowledge, there are no studies evaluating the presence of CTCs prior to surgery in the differential diagnosis of indeterminate adnexal masses. Therefore, we performed this study to evaluate the detection of CTCs by a newly developed platform in the differential diagnosis of adnexal masses.

## RESULTS

### Patient characteristics

Of 87 patients who presented with an adnexal mass, 43 (49.4 %), 13 (14.9 %), and 31 (35.7 %) were pathologically diagnosed with a benign mass, borderline malignant mass, and cancer, respectively. The median age of the population was 47 years (21–78 years). Patients with cancer were older than those with benign tumors (median [range]: 45 years [21–74 years] vs. 57 years [24–77 years]; p = 0.002). Preoperative diagnostic markers had higher mean values in cancer than in benign tumors, including serum CA-125 levels (176.7 U/mL ± 429.3 U/mL vs. 1539.4 U/mL ± 2278.0 U/mL; p = 0.002), ROMA (10.8 % ± 17.4 % vs. 63.0 % ± 36.5 %; p < 0.001), and RMI (642.7 ± 1807.5 vs. 4630.7 ± 7632.6; p = 0.008) (Table 1). These significant differences were maintained when dichotomized into ‘within normal range’ and ‘abnormal’ based on designated cut-off values. On preoperative CT/MRI, findings suspicious of cancer were more significantly associated with a pathologic diagnosis of malignancy (p < 0.001). Tumor size >10 cm (p = 0.024) and moderate to severe ascites on preoperative CT/MRI (p <0.001) were also significantly associated with cancer. For 22 healthy normal controls, the median age was 51.5 years ranging from 46 to 55 years. Mean values of ROMA and serum CA-125 levels were 6.7% ± 2.9% and 13.8 U/mL ± 5.3 U/mL, respectively (data not shown).

**Table 1:** Patient characteristics of study population with adnexal tumor

Characteristic	Total (n=87)	Benign (n=43)	BOT (n=13)	Cancer (n=31)	P*
Age (yr), median (range)	47 (21-78)	45 (21-74)	47 (41-78)	57 (24-77)	0.002
≤47	46 (52.9)	28 (65.1)	7 (53.8)	11 (35.5)	0.012
>47	41 (47.1)	15 (34.9)	6 (46.2)	20 (64.5)	
Preoperative serum CA-125 (U/ml)	646.9±1533.1	176.7±429.3	73.9±144.4	1539.4±2278.0	0.002
≤35	31 (35.6)	17 (39.5)	9 (69.2)	5 (16.1)	0.030
>35	56 (64.4)	26 (60.5)	4 (30.8)	26 (83.9)	
Preoperative ROMA (%)	30.6±35.5	10.8±17.4	17.1±17.6	63.0±36.5	<0.001
Within normal range	37 (43.0)	27 (64.3)	5 (38.5)	5 (16.1)	<0.001
Abnormal†	49 (57.0)	15 (35.7)	8 (61.5)	26 (83.9)	
Preoperative RMI	2019.0±5106.4	642.7±1807.5	237.6±454.9	4630.7±7632.6	0.008
≤200	49 (57.0)	27 (64.3)	10 (76.9)	12 (38.7)	0.030
>200	37 (43.0)	15 (35.7)	3 (23.1)	19 (61.3)	
Preoperative CT or MRI					<0.001
Benign	26 (29.9)	23 (53.5)	2 (15.4)	1 (3.2)	
r/o borderline malignancy	15 (17.2)	9 (20.9)	6 (46.2)	0	
r/o cancer	46 (52.9)	11 (25.6)	5 (38.5)	30 (96.8)	
Laparoscopic operation					<0.001
No	58 (67.4)	21 (48.8)	9 (69.2)	28 (93.3)	
Yes	28 (32.6)	22 (51.2)	4 (30.8)	2 (6.7)	
Tumor size (cm)	15.0±7.1	8.4±5.1	15.0±7.1	10.5±5.8	0.118
≤10	54 (63.5)	33 (78.6)	5 (38.5)	16 (53.3)	0.024
>10	31 (36.5)	9 (21.4)	8 (61.5)	14 (46.7)	
Ascitesǂ					<0.001
Absence	69 (79.3)	39 (90.7)	13 (100)	17 (54.8)	
Presence	18 (20.7)	4 (9.3)	0	14 (45.2)	
Tumor histology					<0.001
Serous	24 (27.6)	4 (9.3)	3 (23.1)	17 (54.8)	
Non-serous	63 (72.4)	39 (90.7)	10 (76.9)	14 (45.2)	
FIGO stage					-
I	19 (43.2)	NA	13 (100)§	6 (19.4)	
II	4 (9.1)	NA	0	4 (12.9)	
III, IV	21 (47.7)	NA	0	21 (67.7)	

Values are presented as number (%) or mean±standard deviation.

*Benign vs. cancer.

†Abnormal ROMA criteria: ≥7.4% (premenopause) and ≥25.3% (postmenopause).

ǂModerate to severe ascites on preoperative CT or MRI.

§Incomplete staging.

BOT, borderline ovarian tumor; CT, computed tomography; CTC, circulating tumor cell; FIGO, International Federation of Gynecology and Obstetrics; MRI, magnetic resonance imaging; NA, not available; RMI, risk of malignancy index; ROMA, risk of ovarian malignancy algorithm; r/o rule out.

In a subgroup analysis of stage I and II cancers vs. benign tumors, the significant differences in age, preoperative serum CA-125 level, and RMI disappeared. The mean difference in preoperative ROMA was significant (10.8 % ± 17.4 % vs. 43.1 % ± 40.0 %; p = 0.032), but patients with abnormal ROMA > reference value were not different between benign vs. stage I and II cancer (Table 2). Although abnormal preoperative CT/MRI findings (p = 0.001) and tumor size >10 cm (p = 0.001) showed significant associations with early stage cancers, there was no significant difference in ascites between benign masses and early stage cancers. Further analysis of stage I vs. benign tumors showed similar findings; however, in this comparison, laparoscopic operation (p=0.194) and mean preoperative ROMA (10.8% ± 17.4% vs. 19.1% ± 21.9%; p = 0.299) were no longer significantly associated with cancer (Table 3).

**Table 2:** Characteristics for patients with benign vs. early-stage ovarian cancer (n=53)

Characteristic	Benign (n=43)	Cancer, stage I and II (n=10)	P
Age (yr), median (range)	45 (21-74)	52.5 (24-73)	0.910
≤46	25 (58.1)	5 (50.0)	0.730
>46	18 (41.9)	5 (50.0)	
Preoperative serum CA-125 (U/ml)	176.7±429.3	652.3±1199.3	0.246
≤35	17 (39.5)	3 (30.0)	0.725
>35	26 (60.5)	7 (70.0)	
Preoperative ROMA (%)	10.8±17.4	43.1±40.0	0.032
Within normal range	27 (64.3)	3 (30.0)	0.075
Abnormal†	15 (35.7)	7 (70.0)	
Preoperative RMI	642.7±1807.5	4860.7±10919.9	0.254
≤200	27 (64.3)	5 (50.0)	0.480
>200	15 (35.7)	5 (50.0)	
Preoperative CT or MRI			0.001
Benign	23 (53.5)	1 (10.0)	
r/o borderline malignancy	9 (20.9)	0	
r/o cancer	11 (25.6)	9 (90.0)	
Laparoscopic operation			0.031
No	21 (48.8)	9 (90.0)	
Yes	22 (51.2)	1 (10.0)	
Tumor size (cm)	8.4±5.1	13.1±5.6	0.013
≤10	33 (78.6)	2 (20.0)	0.001
>10	9 (21.4)	8 (80.0)	
Ascitesǂ			0.114
Absence	39 (90.7)	7 (70.0)	
Presence	4 (9.3)	3 (30.0)	

Values are presented as number (%) or mean±standard deviation.

*International Federation of Gynecology and Obstetrics (FIGO) stage I and II.

†Abnormal ROMA criteria: ≥7.4% (premenopause) and ≥25.3% (postmenopause).

ǂModerate to severe ascites on preoperative CT or MRI.

CT, computed tomography; MRI, magnetic resonance imaging; RMI, risk of malignancy index; ROMA, risk of ovarian malignancy algorithm; r/o rule out.

**Table 3:** Characteristics for patients with benign vs. stage I ovarian cancer (n=49)

Characteristic	Benign (n=43)	Cancer, stage I (n=6)	p
Age (yr), median (range)	45 (21-74)	52.5 (24-73)	0.713
≤46	25 (58.1)	4 (66.7)	>0.999
>46	18 (41.9)	2 (33.3)	
Preoperative serum CA-125 (U/ml)	176.7±429.3	53.1±65.7	0.677
≤35	17 (39.5)	3 (50.0)	
>35	26 (60.5)	3 (50.0)	
Preoperative ROMA (%)	10.8±17.4	19.1±21.9	0.299
Within normal range	27 (64.3)	3 (50.0)	0.658
Abnormal†	15 (35.7)	3 (50.0)	
Preoperative RMI	642.7±1807.5	642.7±1807.5	0.510
≤200	27 (64.3)	5 (83.3)	0.648
>200	15 (35.7)	1 (16.7)	
Preoperative CT or MRI			0.016
Benign	23 (53.5)	1 (16.7)	
r/o borderline malignancy	9 (20.9)	0	
r/o cancer	11 (25.6)	5 (83.3)	
Laparoscopic operation			0.194
No	21 (48.8)	5 (83.3)	
Yes	22 (51.2)	1 (16.7)	
Tumor size (cm)	8.4±5.1	8.4±5.1	0.015
≤10	33 (78.6)	1 (16.7)	0.006
>10	9 (21.4)	5 (83.3)	
Ascitesǂ			>0.999
Absence	39 (90.7)	6 (100)	
Presence	4 (9.3)	0	

Values are presented as number (%) or mean±standard deviation.

†Abnormal ROMA criteria: ≥7.4% (premenopause) and ≥25.3% (postmenopause).

ǂModerate to severe ascites on preoperative CT or MRI.

CT, computed tomography; MRI, magnetic resonance imaging; RMI, risk of malignancy index; ROMA, risk of ovarian malignancy algorithm; r/o rule out.

### Diagnostic performance of circulating tumor cells in differentiating cancer from benign tumor: all stage vs. early-stage cancer

Median CTC count was 1 ranging from 0 to 23. Forty-nine (56.3%) cases had at least one CTC found in preoperative peripheral blood: 19/43 (44.2%) benign, 10/10 (100%) early-stage, and 14/21 (66.7%) advanced-stage cancer. Of 22 normal controls, there was only one (4.5%) who had CTC in her blood sample (data not shown). Mean CTC counts of cancer patients were not significantly different from those of patients with benign tumors irrespective of stage (benign vs. early-stage cancer vs. advanced-stage cancer, 1.5 ± 3.6 vs. 2.0 ± 0.7 vs. 1.8 ± 1.8) (Table 4).

**Table 4:** Preoperative circulating tumor cells of study population with adnexal tumor

Characteristic	Total (n=87)	Benign (n=43)	Borderline malignancy (n=13)	Cancer, early-stage* (n=10)	Cancer, advanced- stage (n=21)	P†
Preoperative CTC count	1.6±2.8	1.5±3.6	1.2±2.0	2.0±0.7	1.8±1.8	0.647/0.725
Preoperative CTC						0.001/0.091
Absence	38 (43.7)	24 (55.8)	7 (53.8)	0	7 (33.3)	
Presence	49 (56.3)	19 (44.2)	6 (46.2)	10 (100)	14 (66.7)	

Values are presented as number (%) or mean±standard deviation.

*International Federation of Gynecology and Obstetrics (FIGO) stage I and II.

†Benign vs. cancer, early-stage/advanced-stage.

CTC, circulating tumor cell.

**Figure 1:**
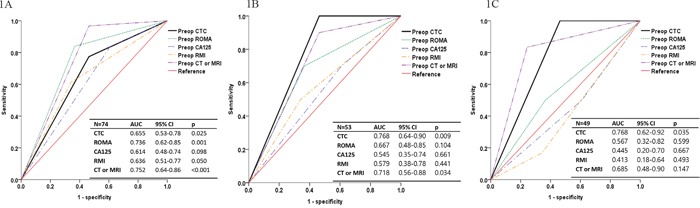
Receiver operating characteristic curves of preoperative diagnostic methods including circulating tumor cell (CTC) detection in the differential diagnosis of adnexal mass **(A)** Benign vs. all stage cancer (n = 74). **(B)** Benign vs. stage I–II cancer (n = 53). **(C)** Benign vs. stage I cancer (n = 49). AUC, area under the curve; CA-125, cancer antigen-125; CT, computed tomography; RMI, risk of malignancy index; ROMA, risk of ovarian malignancy algorithm.

Table 5 and Figure 1 show the diagnostic performance of the various modalities of preoperative differential diagnosis of adnexal masses (benign vs. cancer, excluding borderline malignancy). For benign vs. all stage cancer (n = 74), the sensitivity and specificity of CTC detection were 77.4% and 55.8%, respectively (i.e., false negative rate, 22.6%; false positive rate, 44.2%). McNemar's test showed that the sensitivities and specificities of ROMA (83.9% and 64.3%, respectively), CA-125 (83.9% and 39.5%, respectively), RMI (61.3% and 64.3%, respectively), and CT/MRI (96.8% and 74.4%, respectively) were not significantly different from those obtained by CTC detection. The diagnostic accuracy of CTC detection, ROMA, CA-125, RMI, and CT/MRI was 64.9%, 71.6%, 58.1%, 62.2%, and 83.8%, respectively. Figure 1A shows the receiver operating characteristic (ROC) curves for various preoperative diagnostic methods in the differential diagnosis of adnexal masses (benign vs. all stage cancer). The area under the curve (AUC) (95% CI; p value) for CTC detection, ROMA, CA-125, RMI, and CT/MRI was 0.655 (0.53–0.78; 0.025), 0.736 (0.62–0.85; 0.001), 0.614 (0.48–0.74; 0.098), 0.636 (0.51–0.77; 0.050), and 0.752 (0.64–0.86; < 0.001), respectively.

**Table 5:** Diagnostic performance of preoperative modalities evaluating adnexal mass (benign vs. cancer excluding borderline malignancy)

	Sensitivity	Specificity	Accuracy	P_sens_/P_spec_*
Benign vs. all stage cancer				
CTC	77.4%	55.8%	64.9%	
ROMA	83.9%	64.3%	71.6%	0.727/0.541
CA-125	83.9%	39.5%	58.1%	0.754/0.210
RMI	61.3%	64.3%	62.2%	0.267/0.503
CT or MRI	96.8%	74.4%	83.8%	0.070/0.152
Benign vs. stage I and II cancer				
CTC	100%	55.8%	64.2%	
ROMA	70.0%	63.4%	65.4%	- /0.405
CA-125	70.0%	39.0%	45.3%	- /0.286
RMI	50.0%	65.9%	61.5%	- /0.503
CT or MRI	90.0%	53.7%	60.4%	- /0.189
Benign vs. stage I cancer				
CTC	100%	55.8%	61.2%	
ROMA	50.0%	63.4%	62.5%	- /0.405
CA-125	50.0%	39.0%	40.8%	- /0.286
RMI	16.7%	65.9%	58.3%	- /0.503
CT or MRI	83.3%	53.7%	57.1%	- /0.189

*McNemar test for comparison of sensitivity and specificity with those of CTC, respectively. P value for the comparison of sensitivity in benign vs. early stage cancer could not be calculated because all cancer cases had CTC positive results, that is, sensitivity of CTC 100%.

RMI=U x M x CA-125, U, ultrasound score, is scored 1 point for each of the following characteristics: multilocular cyst, solid areas, metastases, ascites and bilateral lesions. U=0 (for score of 0), U=1 (for score of 1), and U=3 (for score of 2-5). M, menopausal status, is scored as 1 (premenopause) and 3 (postmenopause), which was defined as no period for more than 1 year or age >50 who has had a hysterectomy.

Abnormal criteria: ≥1 for CTC; ≥7.4% (menopause) and ≥25.3% (postmenopause) for ROMA, >35 U/ml for serum CA-125 level; >200 for RMI; report as rule out borderline malignancy or cancer for CT.

CT, computed tomography; CTC, circulating tumor cell; MRI, magnetic resonance imaging; RMI, risk of malignancy index; ROMA, risk of ovarian malignancy algorithm.

For benign vs. stage I and II cancer (n = 53), the sensitivity of CTC detection was 100% (zero false negative rate), bettering the sensitivities of the other modalities (ROMA, 70.0%; CA-125, 70.0%; RMI, 50.0%; and CT/MRI, 90.0%). P values for the comparison of sensitivities of the other modalities with that of CTC detection could not be calculated as all of the cancer cases had positive CTC results. The specificities of ROMA (64.3%), CA-125 (39.5%), RMI (64.3%), and CT/MRI (53.5%) were not significantly different from that of CTC detection (55.8%). Diagnostic accuracy of CTC detection, ROMA, CA-125, RMI, and CT/MRI was 64.2%, 65.4%, 45.3%, 61.5%, and 60.4%, respectively. Figure 1B shows ROC curves for the various preoperative diagnostic methods in the differential diagnosis of adnexal masses (benign vs. stage I and II cancer). The AUC (95% CI; p value) for CTC detection, ROMA, CA-125, RMI, and CT/MRI was 0.768 (0.64–0.90; 0.009), 0.667 (0.48–0.85; 0.104), 0.545 (0.35–0.74; 0.661), 0.579 (0.38–0.78; 0.441), and 0.718 (0.56–0.88; 0.034), respectively.

For benign vs. stage I cancer (n = 49), the sensitivity and specificity of CTC detection did not change from stage I and II cancer (100% and 55.8%, respectively). However, the sensitivities of the other modalities were decreased, although this was not statistically significant. There was no change in specificity for the other modalities. ROC curves showed that the AUC of the modalities other than CTC were decreased, and the curves for preoperative CA-125 and RMI reversed (Figure 1C). The AUC (95% CI; p value) for CTC detection, ROMA, CA-125, RMI, and CT/MRI was 0.768 (0.62–0.92; 0.035), 0.567 (0.32–0.82; 0.599), 0.445 (0.20–0.70; 0.667), 0.413 (0.18–0.64; 0.493), and 0.685 (0.48–0.90; 0.147), respectively. Preoperative CTC detection was the only modality that had a significant difference between the curve and reference line (p = 0.035).

Including borderline ovarian tumors (BOT) in the comparisons decreased the sensitivity of CTC detection (benign vs. BOT and stage I and II cancer, 69.6%; benign vs. BOT and stage I cancer, 63.2%). The AUC of ROC curves of CTC detection also decreased to 0.616 (p = 0.125) for early stage and 0.584 (p = 0.298) for stage I cancer. In both comparisons including BOT, CT/MRI imaging was the only preoperative diagnostic modality showing a significant AUC of ROC curves (CT, 0.703, p = 0.007; MRI, 0.689 p = 0.019; data not shown). These findings suggest that preoperative CTC detection might have the best cancer-detecting performance among the evaluated modalities in distinguishing between benign and stage I ovarian tumors, but this did not extend to benign vs. early stage malignancy including BOT.

### Association of circulating tumor cells with tumor risk factors

Associations between the presence of preoperative CTCs and tumor risk factors were evaluated (Supplementary Table 1). Age >47 years (p = 0.034) and moderate to severe ascites on preoperative CT/MRI (p = 0.009) were significantly associated with presence of CTCs. However, high serum CA-125 level (>35 U/mL), ROMA >reference value, tumor size >10 cm, and CT/MRI findings suspicious of malignancy were not significantly associated with CTCs. Multivariate analysis revealed no independent risk factors for the presence of preoperative CTC among the variables (age >47 years, high serum CA-125 level, and moderate to severe ascites on preoperative CT/MRI), none of which showed a significant (p < 0.2) association with the presence of CTCs in univariate analysis (data not shown).

## DISCUSSION

When an adnexal mass is found in routine ultrasonography without definitive evidence of tumor spreading or distant metastasis and is thought to be borderline or stage I ovarian cancer, it could be challenging for a physician to determine treatment, as an otherwise minimally invasive surgery could be safely performed without significant operation-related complications. Considering practice guidelines which recommend to obtain family history for workup of suspicious pelvic mass and significant proportion (16%) of Korean ovarian cancer patients with a strong family history as well as high prevalence (33%) of *BRCA* mutations in such patients [3, 10], genetic analysis based on the genetic test and family history might be one of the clues favorable for diagnosis of ovarian cancer. Although studies have tried to determine the best method or combination of methods to differentiate ovarian cancer from benign tumors [11, 12], there remains an unmet medical need for differential tools to accurately diagnose early stage ovarian cancer.

Our study demonstrated for the first time that CTCs could be used as a useful diagnostic marker for differentiating ovarian cancer from benign adnexal tumors. Notably, preoperative CTC detection was more sensitive in benign vs. early stage cancer (stage I and II) compared with benign vs. all stage cancer. This improvement remained even in benign vs. stage I cancer. However, serum CA-125, ROMA, RMI, and CT/MRI showed the reverse pattern of diagnostic performance: modest performance in early stage cancer and significantly better performance in all stage cancer excluding BOT. These findings suggest that CTCs might reflect early stage hematogenous metastasis, in contrast to serum CA-125, which reflects advanced-stage peritoneal tumor spread. No significant associations were found between CTCs and serum CA-125 level or ROMA, which supports this hypothesis. Several studies have demonstrated that early hematogenous metastasis in ovarian cancer can occur before peritoneal tumor spread, suggesting that CTCs, so called “liquid tumor biopsies,” could be a feasible method of detection [13–16]. Many relevant studies have reported the presence of CTCs in disease predominantly confined to the abdomen [13]. Fehm et al. observed hematogenous dissemination of isolated tumor cells in stage I ovarian cancer, which implies that single tumor cells might acquire the potential to disseminate to extraperitoneal sites very early in ovarian carcinogenesis [14, 15]. Another report showed that ovarian CTCs implanted and grew in the omentum preferentially and subsequently spread to other peritoneal surfaces using a parabiosis model [16], suggesting that hematogenous metastasis could be an important mode of ovarian cancer metastasis, including intraperitoneal seeding.

Serum CA-125, the best performing single tumor marker so far, is known to be normal or only marginally elevated in approximately 20% of ovarian cancers, especially in early stage disease [12]. Moreover, CA-125 is also elevated in several benign gynecologic and non-gynecologic diseases including endometriosis, adenomyosis, and pelvic inflammatory disease. Recently, Richards et al., in a prospective study, reported that women with stage I ovarian cancer had a higher human epididymis protein 4 (HE4) level compared with those with benign pathology (p = 0.025) [17]. They also showed that the AUC of ROC curves of HE4 was higher than that of CA-125 in all women, with better specificity (p = 0.045). RMI, the most widely used tool for the detection of ovarian cancer, is currently the most accurate tool for stratifying patients into high and low risk groups, with 81% to 92% sensitivity and 82% to 85% specificity [12]. However, some authors insist that ROMA, by combining CA-125 and HE4 together, has better diagnostic performance than RMI [11], whereas others have failed to show an additional benefit of ROMA compared with HE4 or CA-125 alone [18, 19]. Because there is no specific marker uniformly expressed by all cancer types [20] and CTCs are outnumbered by white blood cells by a factor of at least 10^6^ [21], enrichment and purification of CTCs are critical for CTC detection in collected blood. There are various feasible methods, largely biochemical and physical, for isolating CTCs. Biochemical methods, such as the CellSearch system (Veridex), which was approved by the Food and Drug Administration, use CTC-specific antibody-antigen interactions, including epithelial cell adhesion molecule (EpCAM) [22]. Most studies evaluating the prognostic value of CTCs in ovarian cancer using CellSearch have reported negative results, probably owing to the low number of EpCAM-positive CTCs in ovarian cancer or the downregulation of EpCAM during the epithelial-mesenchymal transition [23, 24]. While biochemical methods show unstable capture efficiencies because of varying expression levels by cancer type, physical methods have shown stable capture efficiency regardless of surface marker expression. Therefore, our study team created a new platform using both physical and biochemical methods [22]. Physically, tapered-slit membrane filters (TSF) with vertical slits with a tapered angle of 2° were primarily used for viable CTC isolation, based on CTC size and deformability. Using this TSF platform, about 90% of the cancer cells were captured at a sample flow rate of 5ml/hour, which was 33.3 times faster than previous filters. TSF with a gap that was wide at the entrance and gradually decreased with depth was shown to provide minimal cell stress and reduce 82.14% of the stress generated in conventional straight-hole filters [22]. Biochemically, our criteria included the expression of EpCAM and/or cytokeratin (CK). Finally, morphologic criteria were used for confirming genuine CTCs. With these criteria, we minimized the possibility of missing CTCs, a common problem in EpCAM-only methods.

There are a few limitations to our study. First, the small sample size lowers the power of statistical analysis. Second, the specificities of the evaluated methods were lower than expected, which might be associated with factors related to the study population, because low specificities were observed for all evaluated methods. Therefore, we mainly focused on the sensitivity at a fixed specificity level for evaluating diagnostic performance. However, the low specificity of preoperative CTC detection, that is, its high false positive rate, was not likely owing to our CTC detection method. Lastly, not including a family history in the case report form could be a disadvantage of our study given that the practice guidelines for the management of ovarian cancer address that the initial step is to investigate the family history.

In conclusion, our study findings suggest that preoperative CTC detection could have a substantial role in differentiating early stage cancer from benign adnexal masses, where other commonly used diagnostic methods are not as competent as expected. Nonetheless, the definitive role of CTC in the clinical settings is to be determined, particularly in the field of differential diagnosis of pelvic masses. Diagnostic performance of the CTC detection method using a combination of TSF platform and surface marker expression with confirmatory morphologic criteria should be validated in further studies with a larger sample size.

## MATERIALS AND METHODS

### Patient data and blood sample collection

A total of 87 women with an indeterminate adnexal mass who were scheduled to undergo surgery at Seoul National University Bundang Hospital between May 2015 and April 2016 were prospectively enrolled after getting informed consent. Twenty-two healthy women without no demonstrable adnexal cyst were additionally enrolled for normal control. All of the enrolled patients had received preoperative ROMA, CT, and RMI as standard of care. ROMA was calculated using the following algorithms proposed by Moore et al. [25]:

Premenopausal: PI (predictive index) = -12 + 2.38 × LN(HE4) + 0.0626 × LN(CA-125)

Postmenopausal: PI = -8.09 +1.04 × LN(HE4) + 0.732 × LN(CA-125)

The ROMA-value (predictive value) was then calculated using the following equation:

ROMA (%) = e^PI^/(1+e^P^) × 100

Postmenopausal status was defined as absence of periods for more than 1 yr.

RMI was calculated using the following equation:

RMI = US × menopausal status × serum CA-125 level

US is a quantitative measure of the results of ultrasound score and ranges from 0 to 3. One point is given for each of the following characteristics: multilocular cysts, solid areas, metastases, ascites, and bilateral lesions. US is 0, 1, or 3 for an ultrasound score of 0, 1, or 2–5 points, respectively. Menopausal status is 1 or 3 for premenopause or postmenopause, respectively. MRI was an alternative to CT for the patients who were unable to undergo CT for any reason, such as an allergy to contrast media. Ascites was evaluated on CT/MRI, and a moderate to severe amount of fluid in the abdominal and pelvic cavities was counted as positive for ascites. Patients with a prior malignancy less than 5 years from enrollment were excluded.

While the patient was under general anesthesia, 5 mL of peripheral blood for isolating CTCs was withdrawn from the antecubital vein before the start of surgery. All blood samples were collected in BD Vacutainer® tube and transferred to the Korea Advanced Institute of Science and Technology for identifying and counting CTCs in the blood sample. To avoid cell lysis and destruction during delivery, collection tubes were packed with ice packs in a foam plastic box and delivered within 6 hours after sampling. One week after surgery, diagnosis of the adnexal mass (cancer or benign) and tumor size were confirmed in the final pathological report.

This study was approved by the Institutional Review Board of Seoul National University Bundang Hospital (B-1408/263-003).

### Identification and counting of circulating tumor cells

**Figure 2:**
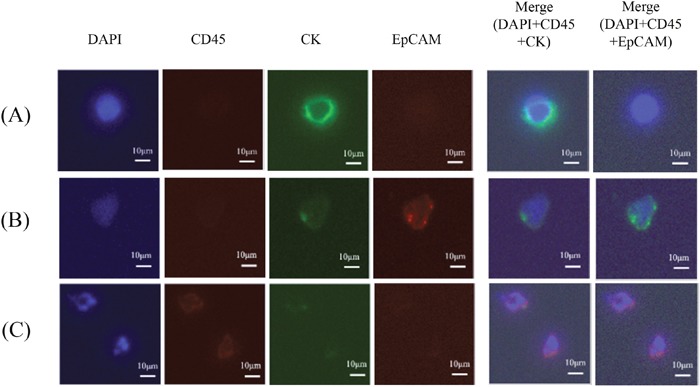
Immunostaining of filtered blood for isolating circulating tumor cells (**A**, DAPI+/CD45-/CK+/EpCAM-; **B**, DAPI+/CD45-/CK+/EpCAM+) and excluding white blood cells (**C**, DAPI+/CD45+/CK±/EpCAM-). The bar represents 10 μm. CD45, cluster of differentiation 45; CK9, cytokeratin 9; DAPI, 4′,6-diamidino-2-phenylindole; EpCAM, epithelial cell adhesion molecule.

CTC isolation and counting were performed using the previously reported TSF platform with optimizations for this work [22]. The TSF isolates CTCs based on their physical properties, such as size and deformability, regardless of their surface protein expression. In addition, its unique design, having a wider cell entrance and gradually narrower slit exits, increases sample flow rate with minimal cell stress, thus achieving rapid, viable CTC isolation from clinical samples. Five milliliters of patient blood was diluted in 10 mL of phosphate-buffered saline (PBS) without any pretreatment and directly processed into the TSF platform under syringe pump. After sample processing, the captured cells were gently released by applying a reverse flow of PBS, and the released cells were mounted onto glass slides by cytocentrifuge (Shandon Cytospin III, Thermo Scientific, Wilmington, DE, USA). The immunostaining protocol was optimized for TSF, and described previously [26]. Briefly, the cell-mounted glass slides were immunostained by fixation, permeabilization, blocking, and immunofluorescent staining. Then, fluorescent images were acquired by fluorescence microscope system (Eclipse Ti, Nikon) and quantified using MetaMorph® software (Molecular Devices, Sunnyvale, CA, USA). All immunofluorescent cells were carefully examined and counted as CTCs considering both staining criteria (4′,6-diamidino-2-phenylindole [DAPI]+, cluster of differentiation 45 [CD45]-, and CK+ or EpCAM+) and morphological criteria, such as bigger size, higher nucleus-to-cytoplasm ratio, and higher degree of irregularity than background blood cells (Figure 2). Staining intensity for positive cases was graded from mild (1+) to severe (3+). The case with intensity 3+ was counted as a positive control. The case was counted as a negative control when no staining intensity was perceived at all.

CTCs were identified and counted by two independent researchers (J Bu, YT Kang), both of whom were blinded to the results of final pathology.

### Assessment of diagnostic performance of CTCs

All of the variables, including presence and mean number of CTCs, were compared between benign and cancerous masses. The association of presence of CTCs with other variables was evaluated for statistical significance. The cut-off values of the preoperative diagnostic tools were as follows: 35 U/mL for serum CA-125 level; 7.4% (premenopause) and 25.3% (postmenopause) for ROMA; and 200 for RMI. By creating ROC curves, sensitivity and specificity of CTC detection for the differential diagnosis of adnexal masses were compared with those of other tools. McNemar's test was used for calculating the statistical significance of each comparison. Otherwise, chi-square test and Student's t-test were used for comparing categorical and numeric variables, respectively. A two-sided p-value of <0.05 was considered statistically significant. SPSS software (version 19.0; SPSS Inc., Chicago, IL) was used for statistical analyses.

## SUPPLEMENTARY MATERIALS TABLE



## Abbreviations

AUC: area under the curve
BOT: borderline ovarian tumor
CD45: cluster of differentiation 45
CK: cytokeratin
CT: computed tomography
CTC: circulating tumor cell
DAPI: 4′,6-diamidino-2-phenylindole
EpCAM: epithelial cell adhesion molecule
HE4: human epididymis protein 4
MRI: magnetic resonance imaging
PBS: phosphate-buffered saline
PET: positron emission tomography
PI: predictive index
RMI: risk of malignancy index
ROC: receiver operating characteristic
ROMA: risk of ovarian malignancy algorithm
TSF: tapered-slit membrane filters.
