# Supplementary material for: Circulating tumor cells in the differential diagnosis of adnexal masses
Source: Oncotarget. 2017 Aug 24;8(44):77195–206. doi: 10.18632/oncotarget.20428 (PMC5652773; doi:10.18632/oncotarget.20428)
Supplement: Supplementary file 1 [file oncotarget-08-77195-s001.pdf]

Circulating tumor cells in the differential diagnosis of adnexal masses

SUPPLEMENTARY MATERIALS

Supplementary Table 1: Associations between presence of preoperative CTC and tumor risk factors (n=87)

Characteristic	Preoperative CTC		p
	Absence	Presence	
Age (yr)			0.034
≤47	25 (54.3)	21 (45.7)	
>47	13 (31.7)	28 (68.3)	
Preoperative serum CA-125 (U/ml)			0.127
≤35	33 (47.8)	36 (52.2)	
>35	5 (27.8)	13 (72.2)	
Preoperative ROMA (%)			0.360
Within normal range	18 (48.6)	19 (51.4)	
Abnormal*	19 (38.8)	30 (61.2)	
Preoperative RMI			0.686
≤200	22 (44.9)	27 (55.1)	
>200	15 (40.5)	22 (59.5)	
Preoperative CT or MRI			0.365
Benign	20 (48.8)	21 (51.2)	
Suspicious of malignancy†	18 (39.1)	28 (60.9)	
Tumor size (cm)			0.400
≤10	26 (48.1)	28 (51.9)	
>10	12 (38.7)	19 (61.3)	
Ascites‡			0.009
Absence	35 (50.7)	34 (49.3)	
Presence	3 (16.7)	15 (83.3)	

*Abnormal ROMA criteria: ≥7.4% (premenopause) and ≥25.3% (postmenopause).

†r/o borderline malignancy or r/o cancer.

‡Moderate to severe ascites on preoperative CT or MRI.

CT, computed tomography; CTC, circulating tumor cell; MRI, magnetic resonance imaging; RMI, risk of malignancy index; ROMA, risk of ovarian malignancy algorithm; r/o rule out.
